# Impact of climatic conditions on radial growth of non-native *Cedrus libani* compared to native conifers in Central Europe

**DOI:** 10.1371/journal.pone.0275317

**Published:** 2023-05-12

**Authors:** Nikola Zsolnay, Anna Walentowitz, Gregor Aas

**Affiliations:** 1 Ecological-Botanical Garden, University of Bayreuth, Bayreuth, Germany; 2 Department of Biogeography, University of Bayreuth, Bayreuth, Germany; Chinese Academy of Sciences, CHINA

## Abstract

Ongoing climate change increasingly affects growth conditions of native conifers such as *Picea abies* (Norway spruce) and *Pinus sylvestris* (Scots pine) in Central Europe. These conifers are primarily cultivated for wood production. To obtain ecologically and economically stable forests, forestry seeks alternative species that might be less prone to novel climatic conditions, such as *Cedrus libani* (Lebanon cedar). We aim at investigating growth responses to climatic factors of *C*. *libani* compared to native *P*. *abies* and *P*. *sylvestris* in Central Europe for 25 years (1994–2019). Growth responses were used as a proxy for tolerance towards climatic stress events, such as heat and drought. Height, diameter at breast height (DBH) and radial increment were measured for 40-year-old tree stands of *C*. *libani* and native conifers. Radial growth responses to selected climate parameters were analysed using bootstrapped correlations with detrended growth index chronologies and growth response indices for drought years (2003, 2012, 2015, 2018). For *C*. *libani*, radial growth was positively correlated with high water availability in late winter and spring, while for *P*. *abies*, February and summer and for *P*. *sylvestris*, July showed such a relationship. *Cedrus libani* exhibited the highest resistance, recovery, and resilience in response to climatic extremes. Against the background of climate change, *C*. *libani* could serve as an alternative conifer species to establish climate-resistant viable forests in Central Europe.

## Introduction

Trees are considered especially sensitive to climate change and the concomitant environmental alterations due to their long generation times [1, 2]. For Central Europe, temperatures are expected to rise, and the frequency and intensity of heatwaves to increase [3, 4]. Accordingly, a decline in vitality, and thus growth, is anticipated for many tree species within this region [5–7]. This negative trend is intensified by an increased vulnerability to pests [8]. *Picea abies* (L.) H. Karst (Norway spruce, Pinaceae) and *Pinus sylvestris* L. (Scots pine, Pinaceae) are widely cultivated and native tree species of great economic importance in Central Europe and impacted negatively by the consequences of global warming [9, 10]. These conifer species are likely to experience reduced productivity with ongoing climate change in Europe and suitable habitat area is projected to decline [11]. However, vital trees and forests are fundamental for preserving ecosystem cycles with long-term dynamics [12] and sustainable forestry.

A potential mitigation and adaptation strategy in forestry in times of climate change is to convert forests into species-rich and resilient mixed stocks that are stable and adjusted to heat and drought events [10, 13, 14]. Part of this strategy is to foster native species that tolerate moderate changes in temperature to preserve potential natural cohabitations. However, in the event of higher mean temperatures, native tree species alone may not be able to maintain forest ecosystem services [7, 15]. This has initiated discussions on additionally cultivating non-native tree species, which are less sensitive to heat and drought events and can be expected to thrive under the future predicted climate in Central Europe [15]. An informed selection and cultivation of suitable non-native trees based on traits and provenance is essential [16]. The selected species are expected to have a high adaptation potential with respect to changing environmental conditions [17]. Therefore, tree species worldwide from regions with a wide-ranging climatic amplitude are being considered for potential use in Central European forestry.

*Cedrus libani* A. Rich (Lebanon cedar, Pinaceae) is an example of a conifer species with expected high potential for adaptation to current and future climatic conditions in Central Europe. Naturally, it distributes in the Taurus Mountains in Southern Turkey, the Lebanon Mountains, and Western Syria [18, 19]. These areas exhibit climatic conditions that can roughly be expected for large areas in Central Europe in the future with ongoing climate change [20].

The objective of our study is to contribute to the evaluation of climate tolerance and adaptability of *C*. *libani* in Central Europe and to compare its performance with the native conifer species *P*. *sylvestris* and *P*. *abies*. We chose a dendrochronological approach to meet the objective, as annual radial growth of trees underlies an intra- and interannual variation that strongly depends on the prevailing climatic conditions. Hence, radial growth is a suitable indicator of the tree’s tolerance toward past extreme climate events [21, 22]. Several studies have analysed the growth and vitality of *C*. *libani* [e.g., 23–27], but to our knowledge, this study is the first to include a direct comparison of growth responses under climate change of *C*. *libani* to native conifers cultivated at the same site in Central Europe. We obtained incremental growth data of *C*. *libani*, *P*. *abies* and *P*. *sylvestris* growing in a confined area in Bayreuth, Germany, located in Central Europe from 1994–2019.

We hypothesise that *C*. *libani* is impacted less by climatic stressors compared to native *P*. *abies* and *P*. *sylvestris*. In detail, the objectives of this study are

1. to detect growth differences between non-native *C*. *libani* and native *P*. *abies* and *P*. *sylvestris*,
2. to identify climatic conditions (temperature and precipitation) influencing the radial growth of each tree species the most,
3. and to detect how strongly radial growth of each tree species was impacted by climatic stress (heat, drought).

## Materials and methods

### Study species and site

**Fig 1:**
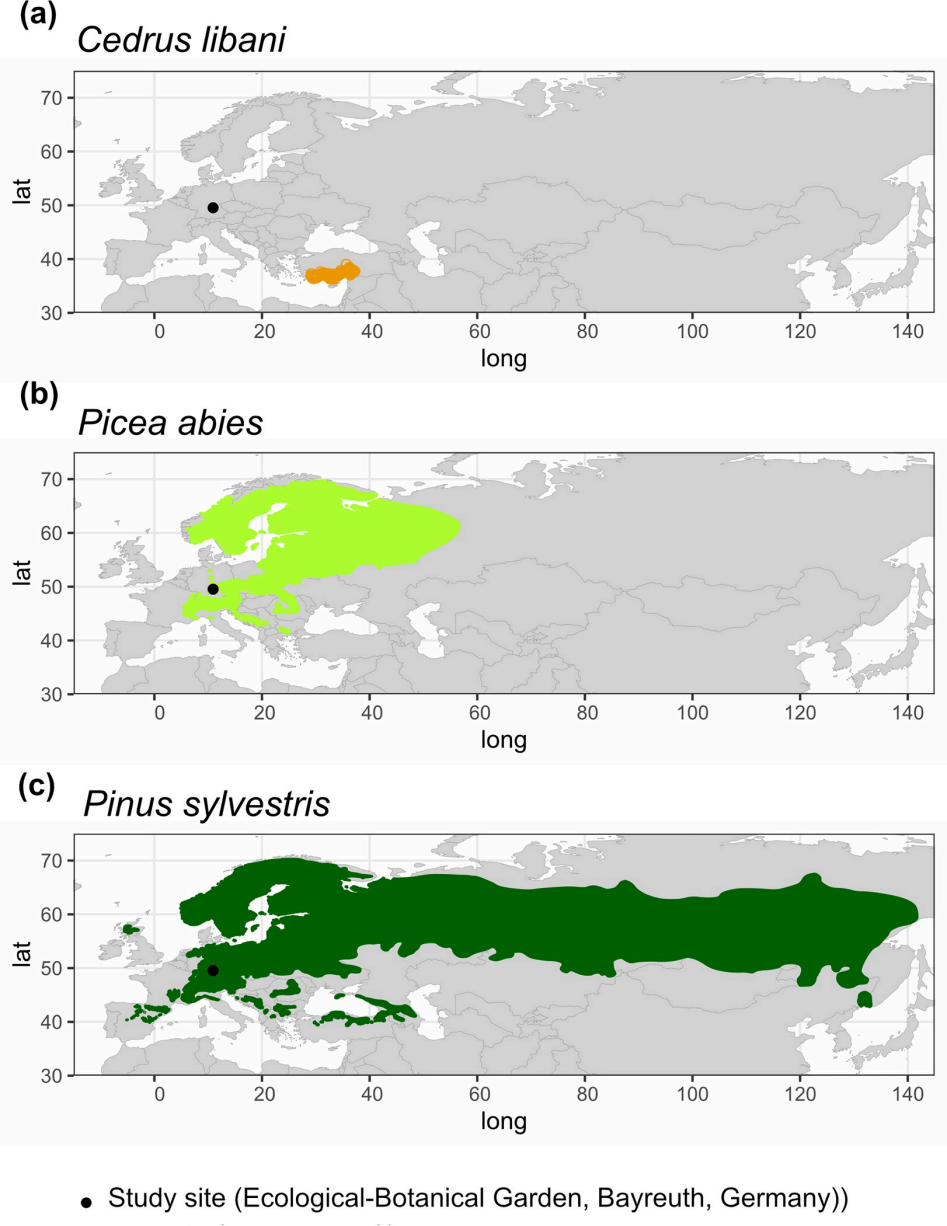
Native distribution of a) *Cedrus libani*, b) *Picea abies* and c) *Pinus sylvestris*. The study site is indicated by a black dot. The distribution maps are based on shape files from [31].

*Cedrus libani* can be subdivided into the subsp. *stenocoma* (Schwarz) Davis originating from the Taurus Mountains of Southern Turkey, and the subsp. *libani* naturally occurring in the Lebanon Mountains and Western Syria [28, 29]. The trees investigated for this study belong to the subsp. *stenocoma*. Within their native range in the Taurus mountains (Fig 1A), *C*. *libani* trees are exposed to a Mediterranean climate with winter precipitation at low elevations near the coast. At higher elevations, the species thrives under a Mediterranean mountain climate, gradually changing into a steppe climate with a precipitation maximum during winter (November - February) and a dry season during summer (June - September) [18]. Annual precipitation sums up to 450 - 1300 mm, and summer droughts of up to 6 months can occur [30]. Annual average temperature ranges between 7.7°C and 15°C [23], and extreme temperatures can reach -35°C in winter and +40°C in summer [19, 30]. In contrast to *C*. *libani*, *P*. *abies* ranges from Central to Northern Europe and *P*. *sylvestris* occurs in Central, Southern, Northern and Eastern Europe, and parts of Central Asia (Fig 1B and 1C).

*Cedrus libani* possesses a deep-reaching taproot or cordate root system [32]. In natural stands in southern Turkey, trees can reach ages up to 800 years, possibly reaching a mean height of 26 m and a mean diameter at breast height (DBH) of 42 cm at the age of 150 years (yield charts of non-thinned stands with excellent site quality) [33]. Trunks are erect, consisting of bright sapwood and a yellowish to red-brown heartwood [19, 33, 34]. The wood is solid, durable, and resistant to decay [19], and thus highly suitable for economically viable wood production. Due to the potential of *C*. *libani* to form stable, productive forest stands in regions experiencing droughts or temperature extremes [35] and its valuable wood, *C*. *libani* is cultivated and used in afforestation projects inside and outside of its region of origin [19, 33].

**Fig 2:**
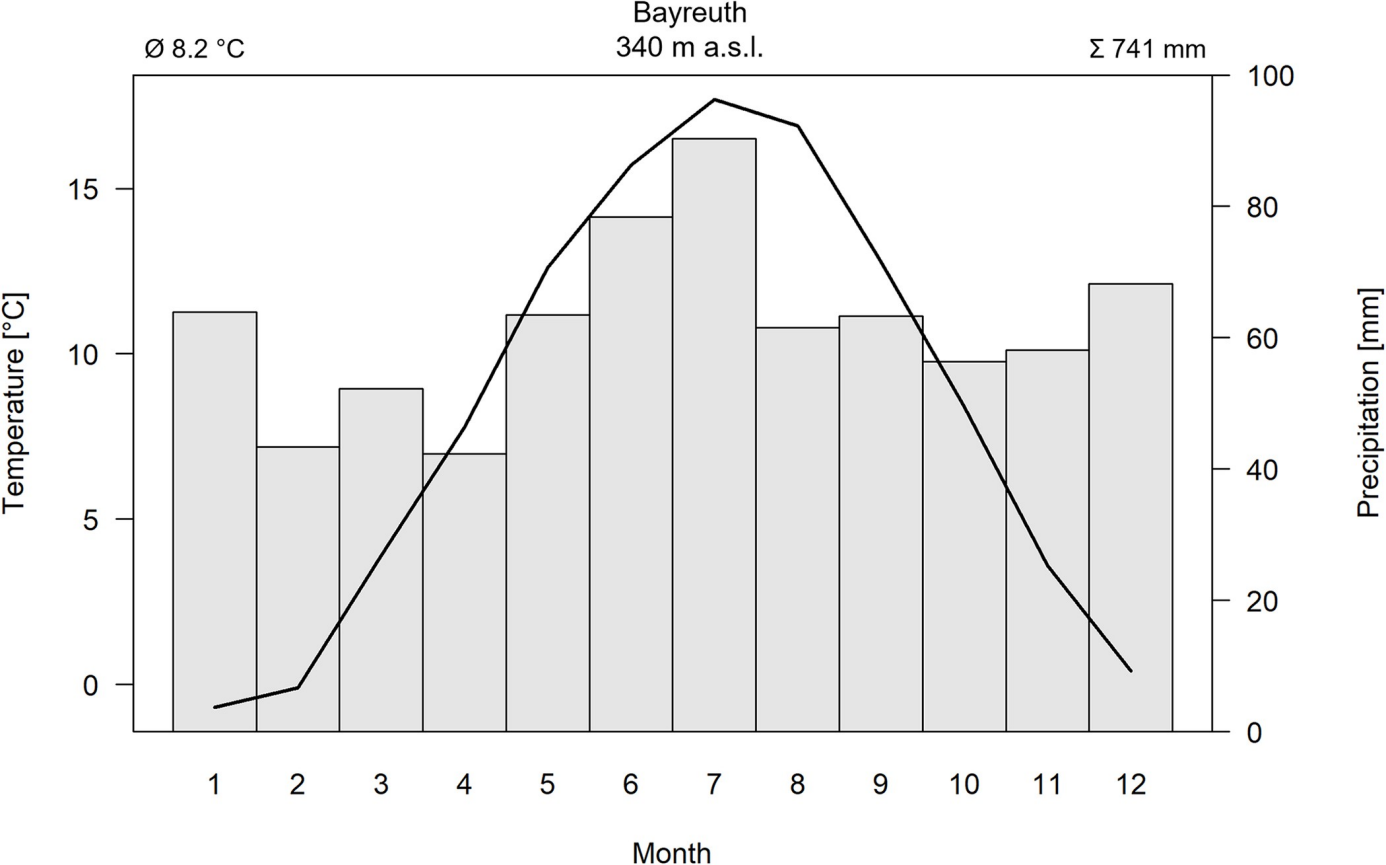
Climate diagram of Bayreuth, Germany, Central Europe, with mean monthly air temperature (black line, C) and monthly precipitation sums (grey bars, mm) for 1980 - 2019 [38]. Mean temperature (top left) and annual precipitation sum (top right) are given.

The study site of 16 ha was located in Central Europe at an elevation of 355 - 370 m a.s.l in the Ecological-Botanical Garden of the University of Bayreuth, Germany (49° 55 ’ 45 ’’ N, 11° 35 ’ 10 ’’ E). The trees were growing within an area of the garden that is not being maintained and experiences neither irrigation, fertilization nor tillage. The site exhibited a transitional climate between oceanic and continental [36, 37] with homogeneous conditions. Mean annual temperature amounted to 8.2°C, and mean annual precipitation summed up to 741 mm (based on data from 1980–2019) (Fig 2). During the vegetation period (April - September), mean temperature amounted to 13.9°C, and mean precipitation summed up to 399 mm [38]. Extreme minimum temperatures of -25°C and maximum temperature of +36.1°C have been recorded [37].

The study site comprised ninety about 40-year-old *C*. *libani* subsp. *stenocoma* trees. Seed material originated from the province of Antalya, Turkey, in the Western Taurus Mountains (1600 - 2000 m a.s.l.) near the town of Elmali. After germination and cultivation in a tree nursery in Germany in 1978, the 2-year-old seedlings were cultivated in the Ecological-Botanical Garden before their final planting [25]. *P*. *abies* and *P*. *sylvestris* grew within the same areas as *C*. *libani*. In total, 27 *C*. *libani*, 17 *P*. *abies* and 13 *P*. *sylvestris* trees of similar age were sampled. All trees had a Kraft crown class of 1–2 [39] and no evident damages. The trees grew on a mixed soil of clay and sandy loam with a shallow top layer of humus that had a pH of 4.4 - 5.3, or on a Luvisol-gley, influenced by stagnant groundwater below 60 cm depth, and with a pH of 4.4 - 5.0 [25].

### Data acquisition and processing

#### Growth data

Trunk diameter at breast height (DBH) and height of the target trees were measured, and tree cores sampled in November 2019. DBH (cm) was measured with a diameter tape and tree height (m) using a laser altimeter rangefinder (Forestry 550, Nikon) and by averaging repeated measurements (three times) for each tree. Two cores per tree were obtained with an increment borer (5 mm diameter, Suunto, Finland) at breast height and a difference in angle of at least 90°. The annual tree ring widths of the cores were measured with a precision of 1/100 mm using TSAP software (TSAP-Win Version 4.69b, Rinntech e. K., Heidelberg, Germany). No permits were needed to conduct the fieldwork as the Ecological-Botanical Garden of the University of Bayreuth is a research facility, allowing investigations of associated researchers.

Differences in mean DBH, height and radial growth between species were tested using a one-way ANOVA and Tukey’s post-hoc test (α  =  0.05). For every tree, the two resulting ring width series were visually synchronised and cross-dated and arithmetically averaged to a mean radial growth series (RGS) per tree. Each RGS was trend-removed, standardised and analysed using the R package dplR [40]. Every RGS mean radial growth series was again arithmetically averaged to radial growth chronologies (RGCs). The availability of at least two RGS per year was set as a requirement for building the RGCs. Mean first-order autocorrelation (Ar1, an estimator of the magnitude of the previous year’s influence on the current year’s growth; [41]) was computed for each RGC.

A cubic-smoothing spline [42] with a frequency cut-off of 50 % at 67 % of the series length was chosen [e.g., 43,44,45] to obtain dimensionless series of growth indices, oscillating around 1.0. Growth indices were calculated as the ratio between the measured radial growth and the fitted values. For each species, a growth index chronology (GIC) was built as Tukey’s biweight robust mean of the standardised growth indices to reduce potential bias caused by extreme values. Mean inter-series correlation (Rbar, an indicator for shared long-term growth variance between the growth indices [46]) and expressed population signal (EPS, a criterion for the strength with which the chronology expresses a common signal in the population from which it was drawn [47]), were calculated for each GIC. To enable comparison with former studies, a threshold of EPS > 0.85 was defined as an acceptable level for a hypothetical noise-free chronology quality [48–50].

Data processing and pre-analysis were performed in R (Version 3.6.2) [51] with the package *varhandle* [52]. Figures and maps were generated in R (Version 3.6.2) [51] as well.

#### Climate data

Nine climate variables and indices related to heat, cold and water supply were selected for climate-growth analysis (Table 1) and are based on measurements from the meteorological station in the Ecological-Botanical Garden, Bayreuth (1994–2019) located at the study site.

**Table 1:** Climate variables and indices related to heat, water supply, and cold and the time scales (monthly, vegetation period or previous winter) considered for subsequent analysis.

Category	Variable	Abbreviation	Time scale
**heat-related **	mean temperature [°C]	T_mean_	monthly, VP, PW
	heat sum [°C] of daily T_mean_ > 20°C	hs	monthly, VP
	number of hot days with T_max_ ≥ 30°C	nhd	monthly, VP
	vapour pressure deficit [hPa]	VPD	monthly, VP
**water supply-related **	precipitation sum [mm]	PPT	monthly, VP, PW
	number of rain days with PPT > 1 mm	nrd	monthly, VP, PW
	standardised precipitation evapotranspiration index for 3 months	spei3	monthly, VP
**cold-related **	number of frost days with T_min_ ≤ 0°C	nfd	monthly, VP, PW
	number of ice days with T_max_ ≤ 0°C	nid	monthly, PW

VP = vegetation period; PW = previous winter.

Temperature, precipitation and relative humidity were measured in time intervals of 10 min and provided as daily minimum, maximum and mean temperature (T_min_, T_max_, T_mean_), daily PPT, and daily potential evapotranspiration (PET). As daily PET was only available from 2001–2019, gaps were filled with data from the meteorological station in Heinersreuth-Vollhof (49° 58 ’ 13 ’’ N, 11° 31 ’ 12 ’’ E; 350 m a.s.l.) of the German Meteorological Service [53]. Additionally, annual averages of T_mean_, and sums of precipitation were calculated monthly for the vegetation period (current April—September) and the previous winter (previous October—current March).

The vapour pressure deficit (VPD in hPa), which provides information on stomatal conductance and transpiration processes in plants, was calculated as the difference between saturation (e_s_) and actual vapour pressure (e_a_) (S1 File) [45, 54]. To compute e_s_ [hPa], temperature values, measured within a 10 min-frequency were used (S2 File) [55]. To calculate e_a_ [hPa], relative humidity values at 10 min resolution were utilized (S3 File) [56]. In order to obtain VPD per month and vegetation period, maximum daily VPD values were arithmetically averaged for the respective time period.

The standardised precipitation evapotranspiration index (spei) was calculated to measure drought severity, taking the effects of precipitation and temperature on drought development into account [57]. It is based on the water balance, resulting from the difference between monthly precipitation and potential evapotranspiration (PET) sums. PET was calculated using the Thornthwaite equation, taking T_mean_ and the degree of latitude into account [58]. To identify extraordinary drought occurrences, a period from 1960 to 2019 was considered. As neither precipitation sum (PPT), nor PET on-site measurements reached this far back, monthly T_mean_ and PPT values from [38] were used to calculate the water balance through 2000. Water balance values based on measurements from [38] and on-site had a high Pearson’s product-moment correlation (r  =  0.93, p < 0.001). However, PPT and PET values from the Ecological-Botanical Garden were used from 2001 onwards, as they could be expected to be more precise. Average monthly and vegetation period spei was calculated at three-month intervals (spei3) [50]. Drought severity was classified as moderate (spei3  = -1.00 to -1.49), severe (spei3  =  -1.50 to -1.99) and extreme (spei3 ≤ -2.00) [59].

Drought stress was furthermore inquired per month, vegetation and previous vegetation period through the number of rain days during the investigation phase. A total of days with precipitation > 1.0 mm was calculated. The same procedure was followed to discover heat and cold stress by obtaining the number of hot days, frost days, and ice days for the subsequent period. The thresholds were set to T_max_ ≥ 30°C, T_min_ ≤ 0°C and T_max_ ≤ 0°C, respectively. Finally, monthly and vegetation period heat sums were computed by calculating the sum of the differences between daily T_mean_ > 20°C and 20°C.

To identify years with climatic stress, extraordinarily high temperatures and extraordinarily little precipitation were considered. Therefore, years with T_mean_ values ≥ 90th percentile during the vegetation period and years with precipitation (PPT) values ≤ 10th percentile during the vegetation period were defined as years with climatic stress events. For confirmation, the 90th/10th percentile of the number of hot days, heat sums, VPD, and number of rain days was furthermore calculated for the vegetation period. All years with climate variable values above or below these percentiles were listed and checked for concordance with the years defined to exhibit climatic stress. The same was done for years with a spei3 value < -1 during the vegetation period [58, 60].

All climate variables were calculated using the R package *dplyr* [61] and Microsoft Excel (Version 2016, Microsoft Corporation). To obtain spei3 and monthly PET, the R package *SPEI* [62] was utilised.

### Climate-growth-analysis

A climate-growth analysis was performed for 1994 - 2019 to assess the influence of specific climate variables on radial growth and the tree species’ tolerance towards climatic stress events. The relationships between the GICs and single monthly and seasonal climate variables were investigated for the period 1994 -2019, using classic correlation functions. As some of the climate variables were not normally distributed, bootstrapped Pearson’s correlation coefficients with a 95 % confidence interval (taking 1000 bootstrap samples from the original distribution of the GICs and climate data) [63] were quantified using the R package *treeclim* [64].

Response indices indicating resistance, recovery and resilience were calculated [65]. Resistance (Rs) quantifies the intensity of a species’ growth depression in the year of the climatic stress event (SE) in comparison to the preceding year(s) (Eq 1). Recovery (Rc) quantifies the development of growth in the year(s) following the climatic stress event in comparison to growth in the year of the event (Eq 2). Resilience (Rl) quantifies a species’ ability to reach growth values of the year(s) preceding the climatic stress event in the year(s) following the event again (Eq 3). Generally, Rc is expected to be lower for species with distinct Rs, as they have a smaller growth depression to recover from.


$$Rs(1)=\frac{{RG}_{SE}}{{RG}_{Pre\;SE}}\;Rc(2)=\frac{{RG}_{SE}}{{mean\;RG}_{Pre\;SE}}$$ Eq 1

$$Rc(1)=\frac{{RG}_{Post\;SE}}{{RG}_{SE}}\;Rc(2)=\frac{{mean\;RG}_{Post\;SE}}{{RG}_{SE}}$$ Eq 2

$$Rl(1)=\frac{{RG}_{Post\;SE}}{{RG}_{Pre\;SE}}\;Rc(2)=\frac{{mean\;RG}_{Post\;SE}}{mean\;{RG}_{Pre\;SE}}$$ Eq 3

The response indices were calculated based on radial growth in the years before and after the climatic stress event, and in the case of sufficient data coverage based on arithmetically averaged growth the two years before and after the event [50, 66]. Including two years in the calculation had the advantage of balancing out favourable and unfavourable climatic conditions for the growth of a particular tree species in the year preceding or following the climatic stress event. All indices were calculated as ratios of the radial growth of every RGS, thus for every tree [10]. For every species, average response index values were computed using the median to minimise the effect of outliers.

Non-parametric Kruskal-Wallis rank sum tests [67] were used to test for differences between species (α  =  0.05), as a normal distribution was not given for some indices. Post-hoc analysis was performed with Dunn’s multiple comparison test (α  =  0.05) with Holm-Šidák adjusted p-values (H_0_ rejected if p ≤ alpha/2). For calculations and figures, the R packages *pgirmess* [68] and *dunn*.*test* [69] were used.

## Results

**Fig 3:**
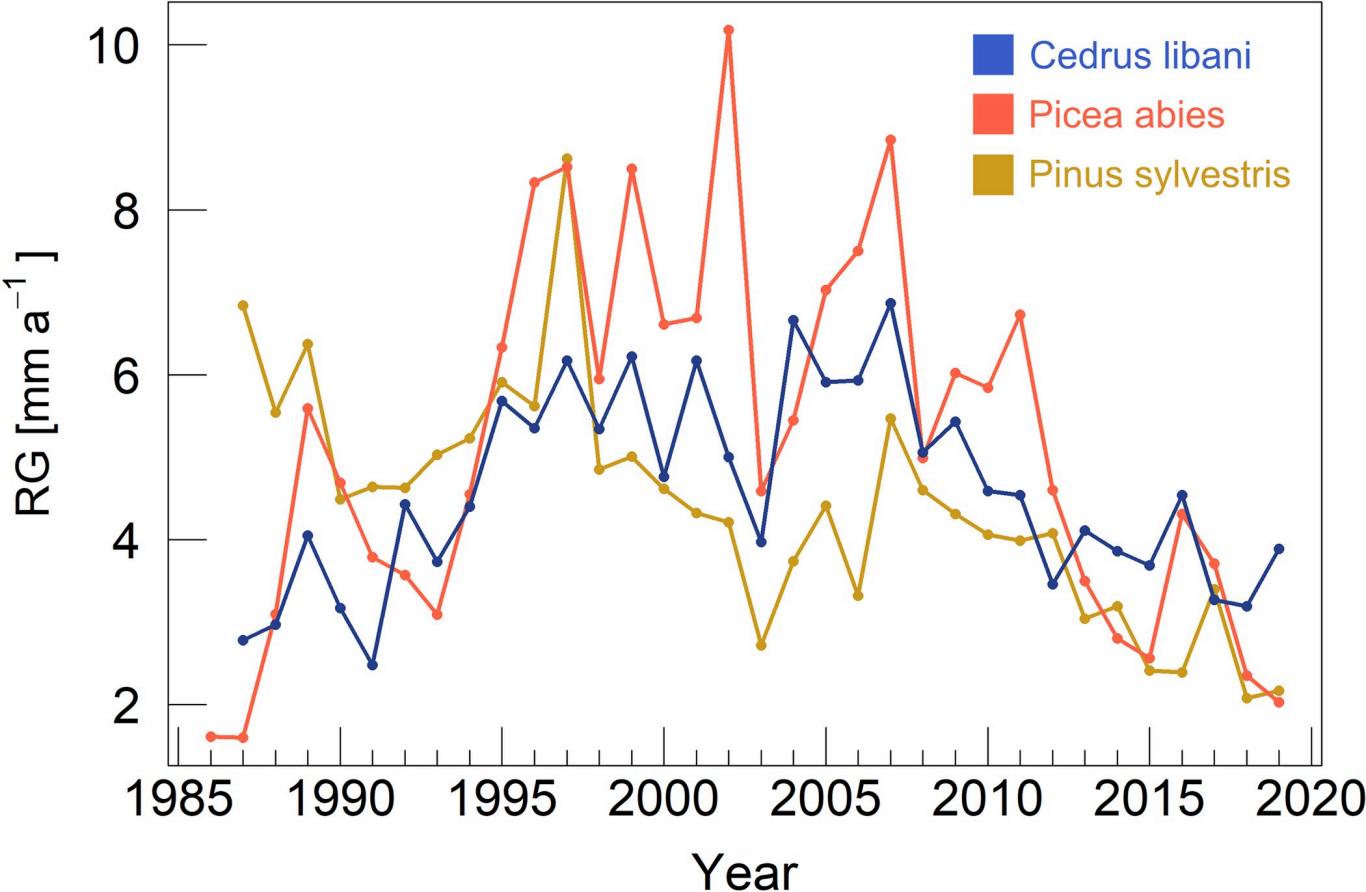
Mean radial growth chronologies (RGCs) of *C*. *libani* (blue), *P*. *abies* (red) and *P*.* sylvestris* (yellow).

Overall, *C*. *libani*, *P*. *abies* and *P*. *sylvestris* showed similar growth patterns. No significant differences in DBH, height, and annual increment rates across species could be found (p_adjusted_ > 0.05; Table 2). Ring widths were widest for *P*. *sylvestris* through 1994, and from then on, the smallest in most years (Fig 3). Between 1995 and 2013, *P*. *abies* showed the highest growth rates, except for 2004, where growth rates of *C*. *libani* exceeded them. From 2014 through 2019, except for 2017, the radial increment of *C*. *libani* was highest.

**Table 2:** Mean DBH (and standard deviation; cm), height (m) and radial growth (mm a^-1^) of *C*. *libani*, *P*. *abies* and *P*. *sylvestris*.

Species	DBH (cm)	Height (m)	Radial Growth (mm a^-1^)
*C*.* libani *	34.3 ± 7.1	15.8 ± 2.1	4.6 ± 1.2
*P*.* abies *	37.0 ± 8.0	17.8 ± 2.3	5.2 ± 2.2
*P*.* sylvestris *	33.9 ± 3.6	15.6 ± 1.1	4.4 ± 1.4

**Fig 4:**
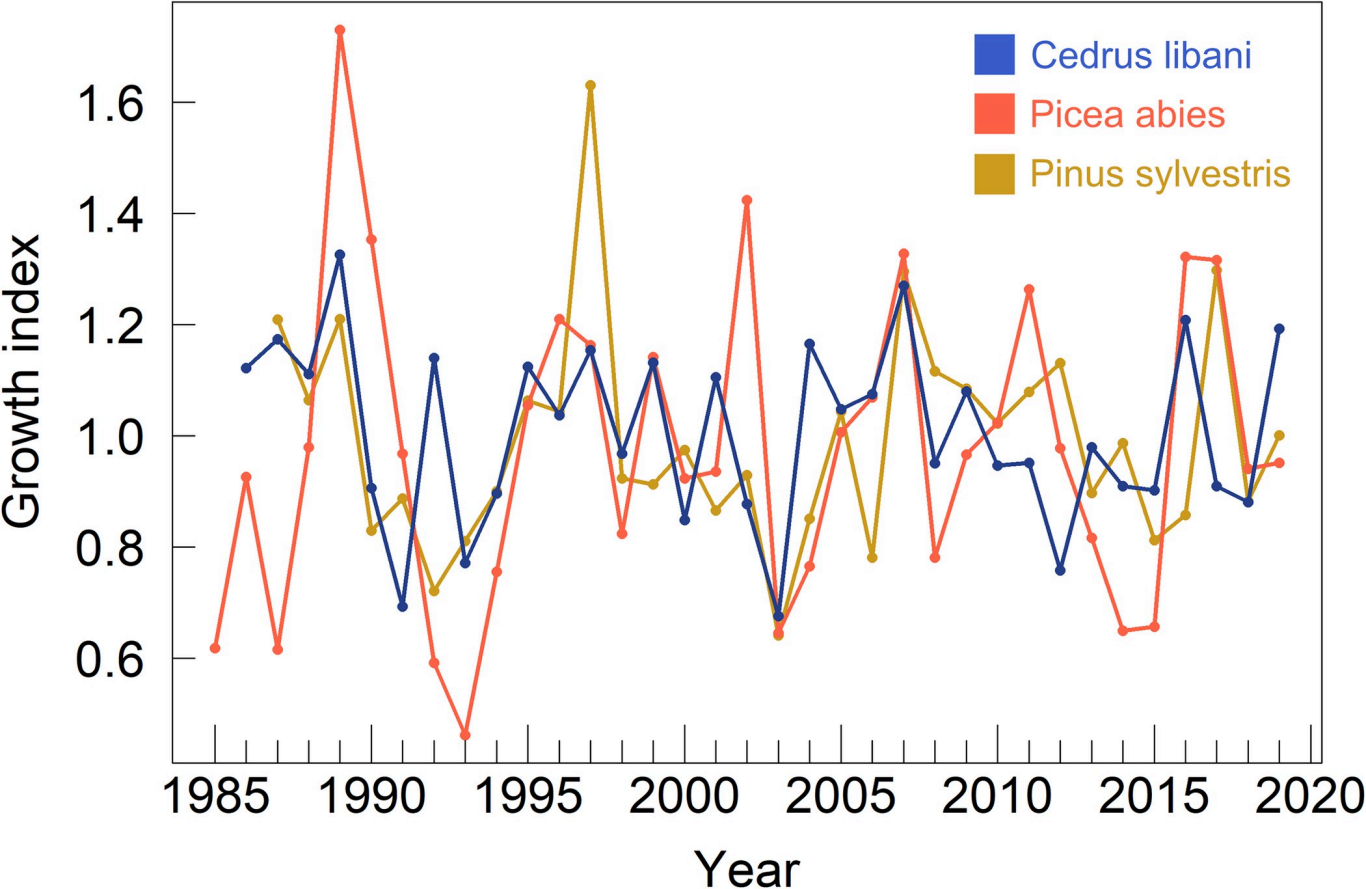
Growth index chronologies (GICs) of *C*. *libani* (blue), *P*. *abies* (red) and *P*. *sylvestris* (yellow).

Radial growth of *C*. *libani* was the most influenced by the previous year as Ar1 values ranged between 0.60 (*C*. *libani*) and 0.55 (*P*. *abies*) (S1 Table). The best agreement across growth indices was found for *P*. *abies* with an Rbar of 0.48. The lowest Rbar was calculated for *C*. *libani* (0.39). All chronologies exhibited EPS values above the conventionally applied threshold of 0.85 [47], and can therefore be regarded as reasonable proxies for climatic signals (Fig 4).

### Climatic stress events

2003 and 2018 were identified as years with climatic stress, as T_mean_ values ≥ the 90th percentile and precipitation values ≤ the 10th percentile during the vegetation period were found. For 2015, a precipitation value ≤ 10th percentile during the vegetation period and for 2019, a T_mean_ value ≥ 90th percentile during the vegetation period were detected.

As an indication for heat stress, values ≥ 90th percentile during the vegetation period were found for the number of hot days in 1994, 2003, 2015, 2018 and 2019, for heat sums in 1994, 2015 and 2018, and for VPD in 2015, 2018 and 2019. Values ≤ 10th percentile were found for the number of rain days in 2003, 2015 and 2018, suggesting water deficiency. According to spei3 values < -1 during the vegetation period, an abrupt moderate drought was detected for 2003 and 2018, a persistent moderate drought for 2012, 2015 and 2019, and a persistent severe drought for 2003 and 2018. The years 1994 and 2019 were not included in the analysis as stress events due to a lack of data in 1993 and 2020 and an adequate water supply throughout the year and the vegetation period in 1994. A detailed description of the different climatic characteristics of each stress event can be found in the S4 File.

### Climate-growth-correlations

Bootstrapped correlation functions between growth index chronologies and specific climate variables revealed species-specific differences in the influence of these variables on tree growth for various time intervals (Table 3).

**Table 3:** Significant (p < 0.05) bootstrapped Pearson’s correlation coefficients (R) for climate-growth-correlations between climate variables and growth index chronologies of the different species for the vegetation period (VP, Apr.-Sep) and single months. Months of the previous year are written in lower case letters and months of the current year in capital letters. Background colours indicate heat-related (red), water supply-related (blue) and cold-related (yellow) climate variables.

Climate variable	*C*. *libani*		*P*. *abies*		*P*. *sylvestris*	
	Month(s)	R	Month(s)	R	Month(s)	R
**T**_**mean**_					VP	-0.33
					MAR	0.38
					JUL	-0.37
					SEP	-0.30
**number of hot days with T**_**max**_ **≥ 0°C**			VP	-0.48	VP	-0.44
			JUL	-0.31	JUL	-0.34
			AUG	-0.49		
**heat sum [°C] of daily T**_**mean**_ **> 20°C**	VP	-0.43	VP	-0.48	VP	-0.47
	AUG	-0.52	JUL	-0.31	JUL	-0.36
			AUG	-0.49	AUG	-0.42
**vapour pressure deficit [hPa] (VPD)**	nov	0.39	JUL	-0.33	JUL	-0.34
	MAR	-0.40				
**precipitation sum [mm]**	FEB	0.44	VP	0.44	JUL	0.66
			FEB	0.59		
			JUN	0.57		
			JUL	0.43		
**number of rain days with PPT > 1 mm**	dec	-0.46	FEB	0.51	JUL	0.62
	FEB	0.46	JUL	0.34		
	JUN	0.44				
**spei3**	MAR	0.51	VP	0.35	JUL	0.42
	APR	0.39	JUL	0.40	AUG	0.51
	MAY	0.37	AUG	0.52	SEP	0.41
**number of frost days with T**_**min**_ **≤ 0°C**			sep	-0.32	MAR	-0.38
					MAY	0.52

Positive correlations (0.34 ≤ Pearson’s r ≤ 0.66, p < 0.05) with water supply-related variables were found for all three species, varying on a temporal scale. For *C*. *libani*, positive correlations with all water supply-related variables (0.37 ≤ Pearsons’s r ≤ 0.51) were found for months in late winter/spring, with February as the most represented month. The overall strongest positive correlation was detected between growth of *C*. *libani* and spei3 in March (Pearson’s r  =  0.51), and rain days in June correlated positively with radial growth. Growth of *P*. *abies* correlated strongest and positive with precipitation in February (Pearson’s r  =  0.59). For that month as well as between June and August, positive correlations with all water supply-related variables were detected (0.34 ≤ Pearson’s r ≤ 0.59) for *P*. *abies*. Correlations for June exhibited the highest values of all summer months for this native conifer species. A strong positive correlation was detected for growth of *P*. *sylvestris* with every single water supply-related variable in July (0.42 ≤ Pearson’s r ≤ 0.66). A high correlation with spei3 was relevant in September (Pearson’s r  =  0.41) for *P*. *sylvestris*.

Heat correlated negatively with the growth of all species (-0.52 ≤ Pearson’s r ≤ -0.31) during the vegetation period and in species-specific months in the summer. For the growth of *C*. *libani*, the overall strongest negative correlation (Pearson’s r  =  -0.52) was detected with the heat sum of daily T_mean_ > 20°C during the vegetation period and in August. Many hot days and a large heat sum during the vegetation period, in July, and especially in August negatively correlated with the radial increment of *P*. *abies* (-0.39 ≤ Pearson’s r ≤ -0.31). The highest number of correlations with heat-related climate variables were found for the growth of *P*. *sylvestris* (-0.47 ≤ Pearson’s r ≤ 0.38). From midsummer through September, and foremost in July, moderate temperatures promoted radial growth of this conifer species. The overall strongest negative correlation with growth of *P*. *sylvestris* was found for heat sums in the vegetation period (Pearson’s r = -0.47).

A negative correlation of growth with the number of frost days could only be detected for *P*. *abies* in previous September and for *P*.* sylvestris* in March. *C*. *libani* did not correlate significantly with any of the cold-related variables.

### Response indices

**Fig 5:**
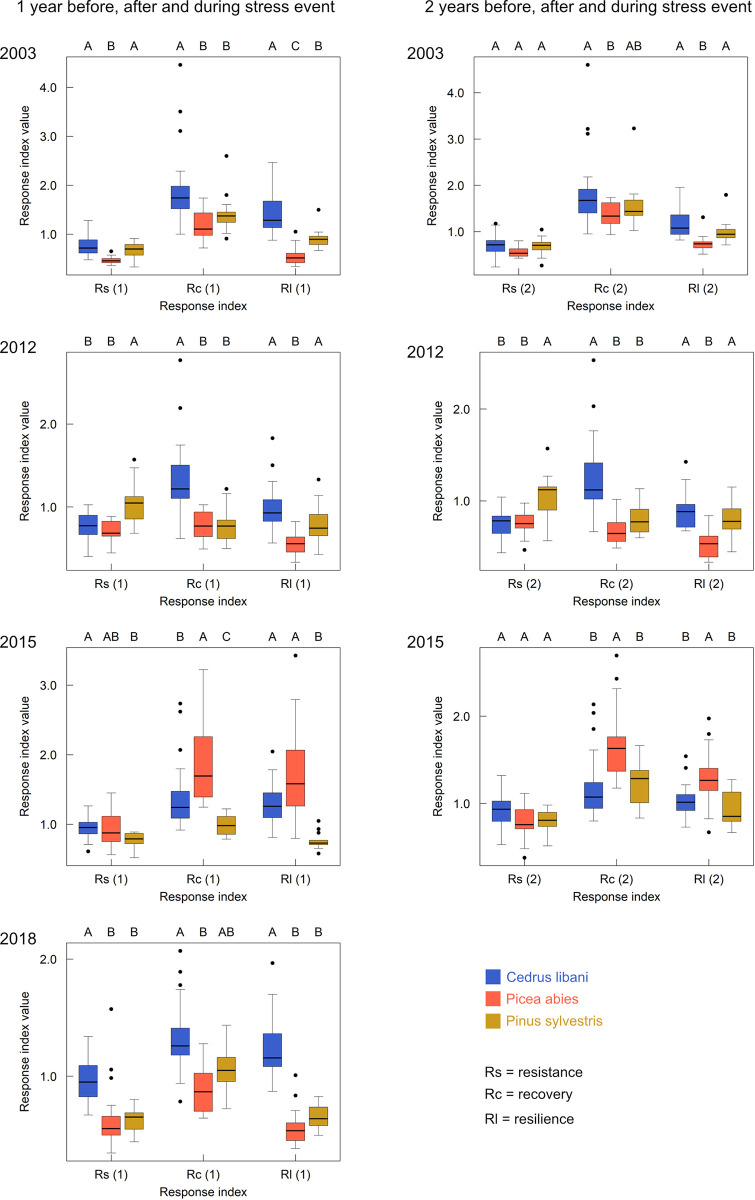
Response indices resistance (Rs), recovery (Rc) and resilience (Rl) for *C*. *libani* (blue), *P*. *abies* (red) and *P*. *sylvestris* (yellow) based on radial growth in the year before, during, and after the climatic stress event in 2003/2012/2015/2018 (left) and based on average radial growth in the two years before/after and in the year of the climatic stress event in 2003/2012/2015 (right). Significant differences across species are indicated by capital letters (Kruskal-Wallis rank sum test with p < 0.001 and df = 3 and Dunn’s multiple comparison test with Holm-Šidák adjusted p-values < 0.05).

**Fig 6:**
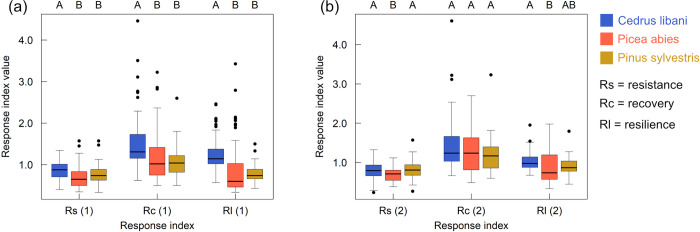
Average response indices resistance (Rs), recovery (Rc) and resilience (Rl) for *C*. *libani* (blue), *P*. *abies* (red), and *P*. *sylvestris* (yellow) (a) based on radial growth in the year before, during, and after a climatic stress event and (b) based on average radial growth in the two years before/after and in the year of a climatic stress event. Average response indices were calculated as the median of those for (a) the SEs 2003, 2012, 2015 and 2018 and (b) the SEs 2003, 2012 and 2015. Significant differences across species are indicated by capital letters (Kruskal-Wallis rank sum test with p < 0.001 and df = 3 and Dunn’s multiple comparison test with Holm-Šidák adjusted p-values < 0.05).

The difference in tolerance towards climatic stress across species was assessed for every single climatic stress event (Fig 5; Table 4) as well as for all such events together, based on average response indices calculated as the median of those for the single events (Fig 6; Table 4; S1 Fig).

**Table 4:** Response indices based on radial growth in the year before, during, and after the climatic stress event (black) and based on average radial growth in the two years before/after and in the year of the climatic stress event (grey), as well as average response index-values (calculated as the median) for each species.

Species	Index	Climatic stress events				Avg. Index-Value
		2003	2012	2015	2018	
***C*.* libani ***	**Rs (1) **	0.72	0.77	0.95	0.95	0.88
***P*.* abies ***		0.46	0.68	0.88	0.55	0.65
***P*.* sylvestris ***	** **	0.70	1.05	0.79	0.65	0.74
***C*.* libani ***	**Rs (2) **	0.72	0.78	0.93		0.79
***P*.* abies ***		0.53	0.75	0.76		0.71
***P*.* sylvestris ***	** **	0.71	1.12	0.81		0.81
***C*.* libani ***	**Rc (1) **	1.74	1.22	1.24	1.26	1.31
***P*.* abies ***		1.11	0.77	1.69	0.87	1.02
***P*.* sylvestris ***	** **	1.37	0.77	0.98	1.05	1.04
***C*.* libani ***	**Rc (2) **	1.68	1.12	1.07		1.24
***P*.* abies ***		1.33	0.64	1.63		1.24
***P*.* sylvestris ***	** **	1.44	0.77	1.28		1.17
***C*.* libani ***	**Rl (1) **	1.29	0.93	1.26	1.16	1.14
***P*.* abies ***		0.52	0.56	1.58	0.54	0.60
***P*.* sylvestris ***	** **	0.90	0.74	0.73	0.64	0.74
***C*.* libani ***	**Rl (2) **	1.08	0.88	1.01		0.97
***P*.* abies ***		0.74	0.53	1.26		0.74
***P*.* sylvestris ***	** **	0.94	0.78	0.85		0.87

Rs =  resistance; Rc  =  recovery; Rl  =  resilience; (1)  =  based on radial growth in one year before/after the climatic stress event; (2)  =  based on average radial growth in two years before/after the climatic stress event.

All species exhibited a depression in relative growth in 2003, followed by an increment in the following year, which did not exceed that of the year 2003 with the exception of *C*. *libani*. The same result was obtained when taking average relative growth within the two preceding and two subsequent years of 2003 into account. *C*. *libani* showed the highest Rs (0.72/0.72), Rc (1.74/1.68) and Rl (1.29/1.08). Relative growth of *P*. *abies* was the most affected.

Except for *P*. *sylvestris*, all species showed a depression in relative growth in 2012, from which only *C*. *libani* recovered in the subsequent year. Nonetheless, no species retrieved growth values equal to or higher than values in the year preceding 2012. Similar results were found when conducting the analysis based on two years. *P*. *sylvestris* exhibited the highest Rs (1.05/1.12), and *C*. *libani* the highest Rc (1.22/1.12) and Rl (0.93/0.88). *P*.* abies* was the most affected.

The two native conifers and *C*. *libani* reacted to the climatic conditions with a growth depression in 2015, from which *C*. *libani* and *P*. *abies* recovered in 2016 to relative growth values exceeding those in 2014. Relative growth further decreased for *P*. *sylvestris*. Considering two previous and subsequent years of 2015, stress tolerance became higher for *P*. *abies* and lower for *P*. *sylvestris* and *C*. *libani*, leading to an overall approximation of the response indices of the species. *C*. *libani* exhibited the highest Rs (0.95/0.93), and *P*. *abies* the highest Rc (1.69/1.63) and Rl (1.58 /1.26).

All three species showed a growth depression in 2018, which was foremost notable for the two native species. *C*. *libani* was the only species that recovered with a relative growth that exceeded the growth in 2017. Hence, stress in 2018 had the smallest effect on *C*. *libani*, which exhibited the highest response index values of 0.95, 1.26 and 1.16. When taking the species’ average tolerance towards all stress events into account, *C*. *libani* was by far the least affected and the only species that on average recovered to relative growth in the year after the stress event and even exceeded growth compared to the year before the stress event (S1 Fig). It exhibited the significantly highest Rs (0.88), Rc (1.31) and Rl (1.14) values. Response indices were the second-highest for *P*. *sylvestris* (0.74, 1.04 and 0.74) and the lowest for *P*. *abies* (0.65, 1.02 and 0.60).

Using response indices based on two years prior and post a stress event led to different results (S1 Fig). Although its value slightly increased, Rs remained the, now significantly, lowest for *P*. *abies* (0.71). It furthermore decreased for *C*. *libani* and increased for *P*.* sylvestris*, leading to similar values (0.79 and 0.81, respectively). Rc became equal for *C*. *libani* and *P*. *abies* (1.24) and increased to 1.17 for *P*. *sylvestris*. Rl was still around 1 and remained the highest for *C*. *libani* (0.97), with, however, a significant difference only to *P*.* abies*. For both native conifers, Rl increased (0.87 *P*. *sylvestris* > 0.74 *P*. *abies*) but remained < 1.

## Discussion

*Cedrus libani* reacted less to climatic stress than the native conifer species *Picea abies* and *Pinus sylvestris*, growing at the same site in Bayreuth, Germany, Central Europe. Overall, all three species had similar growth without significant differences in mean DBH, height and radial growth.

Radial growth of *C*. *libani* was least affected by climatic stress events, followed by *P*. *sylvestris* and *P*. *abies*, respectively. Across years, *C*. *libani* showed the lowest resistance (Rs) to climatic stress in 2003. Together with 2018, the climatic stress event in 2003 can be considered the most severe, with a persistent drought lasting for the entire vegetation period. Additionally, precipitation in February was scarce, assumedly worsening growth conditions in that year for *C*. *libani*. Notwithstanding, that 28 % decline in ring width was less than the strongest decline found for *P*. *sylvestris* (35 % in 2018) and *P*. *abies* (54 % when one year previous to the stress event was considered, 47 % when two years previous to the stress event were considered in 2003). Furthermore, *C*. *libani* recovered best from this stress event, making it across years and species the most resilient in 2003 (Rl  =  1.29/1.08). [25] detected recovery of *C*. *libani* trees to average growth values in the following year of stress events at the study site. In general, *P*. *sylvestris* reacted similarly to the stress events in 2003 as *C*. *libani*. Strongly impacted at first, it exhibited a Rs value comparable to that of *C*. *libani*, followed by its highest recovery and resilience across years. This similar reaction to drought could be attributed to both tree species’ strong stomata control and potentially deep-reaching root system [32]. *P*. *abies*’ substantial reduction in ring width and its incapability to recover within the next two years can be explained by its weak stomata control and shallow root system [9, 70]. A low Rs value (0.62) and no actual resilience (Rl  =  0.78) of *P*. *abies* was also detected by [10], taking eight different sites in Bavaria and response indices based on three years into account.

Higher climatic stress tolerance of *C*. *libani* compared to the two native conifer species was furthermore stressed by the species’ reaction to the stress event in 2018. Rs (0.95) of *C*. *libani* was one of the highest values across years, particularly across species. Additionally, *C*. *libani* was the only species exhibiting actual resilience (Rl  =  1.16). Its stronger impairment of radial growth in 2003 compared to 2018 was relativised when taking growth in two years preceding the stress events into account. As a result, Rs in 2003 stayed the same, but Rs in 2018 dropped to 0.80 as tree rings where wider in 2016 than in 2017. This emphasises the importance of including growth of several years in the calculation of response indices [65]. However, as climatic stress events become more frequent with ongoing climate change, a strict differentiation between years of normal climatic conditions and years with climatic stress to assess how severely tree species are affected will get more challenging in the future, as in the case of the stress event in 2018. Climatic growth conditions in the subsequent year 2019 were highly unfavourable for plant growth, and it is impossible to determine how well the tree species would have recovered from climatic stress in 2018.

The stress event with the most negligible impact on tree growth occurred in 2015. It was characterised by average temperatures and water supply in spring and climatic stress was limited to a summer drought starting in July and accompanied by extremely high temperatures throughout September. In accordance with *C*. *libani*’s adaptation to a low water availability during that time of the year, the species exhibited a Rs of 0.95/0.93 and actual resilience. Interestingly, *P*. *abies* was least affected by this climatic stress event across all years, contrary to its vulnerability to high temperatures and scare water supply in summer, for which literature does not offer an explanation. The observation that *P*. *sylvestris* was overall more affected by the stress event in 2015 can be linked to the climate-growth-correlations obtained. As in 2015 the highest values for heat sum and number of hot days across years were found, they might have provoked a negative mass balance for *P*. *sylvestris* [71]. In accordance, no recovery of *P*. *sylvestris* in the following year was observed in Middle Franconia, Germany [72]. These findings are in accordance with current literature that names drought stress during later stages of the vegetation period to lead to declining vitality in *P*. *sylvestris* [73]. Taking the type and the intensity of climatic stress into account when determining stress events and analysing species’ tolerance is essential. All species demanded an ample water supply, slightly varying in time. Water availability during the vegetation period is one of the most important climatic factors influencing growth of European forests [74, 75]. Interestingly, a high water availability during months prior to the vegetation period, especially in February, was a requirement for strong radial growth of *C*. *libani*. A combination of climatic conditions and the physiology of the species might explain this observation. Firstly, precipitation peaks between November and February at high elevations in its region of origin [18]. *C*. *libani* is thus assumedly adapted to abundant precipitation during that time of the year. Strong stomata control and its ability to store water in its xylem are in support of this explanation [27]. At the study site, the average precipitation sum outside the vegetation period is lower compared to the place where the seeds originate from ([25] based on data from the Southwest Anatolian Forest Research Institute), and lowest in February [38]. Correspondingly, below-average precipitation in February at the study site might constitute a limiting factor for radial growth. Secondly, precipitation at the end of the winter fills up soil water reservoirs of which trees can draw on when xylogenesis starts [76]. Cambial activity, and therefore radial growth, was reported to primarily occur in spring, presenting an adaptation strategy to the summer drought period [77, 78]. Another factor arising in spring and potentially determining final ring width of *C*. *libani*, is its needle cycle. *C*. *libani* only maintains a few needle sets and tends to discard the oldest set in spring. However, if water supply is high and no limitation of stomatal conductance is required, the needle set may be preserved a bit longer, leading to higher CO_2_ assimilation.

Although *C*. *libani* is adapted to low water availability, it likely also profits from a good water supply in the summer by showing increased radial growth. The species’ radial growth correlated significantly with the number of rain days in June at the study site. An optimal maximum precipitation sum of around 425 mm during the vegetation period resulted in best growth performance that could be enhanced furthermore by regular rainfall [32]. In accordance, we observed a larger average ring width of *C*. *libani* at the study site than natural stands, where the annual precipitation sum is only around 100 mm lower and unevenly distributed throughout the year.

Good water supply in February and during the vegetation period (especially in the summer) promoted growth of *P*. *abies*. Similar to *C*. *libani*, humid conditions shortly before the vegetation period fill up soil water reservoirs and are essential for *P*. *abies* growth [76]. Both species also had in common that well-distributed precipitation in June favoured radial growth. However, different to *C*. *libani*, *P*. *abies* depended strongly on a high water availability throughout the summer. Opposite to *C*. *libani*, *P*.* abies* tends to have a shallow root system [70]. Hence, it only has limited access to deep soil water sources, which is especially problematic during drought stress, as it has more difficulties reducing transpiration [9, 79]. Therefore, sufficient precipitation to maintain a relatively moist topsoil layer is required for most of the growing season, particularly when temperature and therefore VPD of the air are high. [80] observed for many days with no or only little precipitation an ongoing shrinkage of *P*. *abies* stems, as transpiration losses could not be compensated by water absorption via the root system. Such reversible decline in stem radius may last up to several weeks [81, 82]. The emphasised importance of a sufficient water supply in June could be explained by the fact that large parts of secondary growth generally typically occur during that month [9, 83].

Similar to *C*. *libani*, the physiological and morphological traits and strategies of *P*. *abies* can be related to conditions in the area of origin. As *P*. *abies* mainly occurs in cool, humid northern or high montane and subalpine areas in Europe and Asia, the species would typically not need a high adaptation to a persistent water shortage. Correspondingly, spruce cultivated beyond its natural distribution range, especially in times of global warming, is susceptible to drought stress [9].

High water-availability in July was crucial for strong radial growth of *Pinus sylvestris*. As this species has higher control over stomatal conductance and thus transpiration than for example *P*. *abies* and a deeper reaching root system, unrestrained water deprivation and cavitation-induced gas emboli are less likely to occur [9, 84]. Hence, *P*. *sylvestris* is more capable of coping with drought, also underlined by significant correlations with water supply-related climate variables, which were mainly constrained to one month. However, if dry climatic conditions occur in addition to water shortages, stomatal closure leads to a reduction of photosynthesis at an early stage of a drought [85, 86], limiting radial growth [87]. [76] detected that abundant precipitation from May to August facilitated growth of *P*. *sylvestris* growing in a temperate forest southeast of Paris, France. Furthermore, they discovered that latewood growth depended on the current growing season’s climatic conditions and soil water supply. For the months May to July, [16] identified a negative impact of a high water deficit on radial growth of *P*. *sylvestris* stands at different sites throughout Europe.

The lack of significant correlations of *C*. *libani*’s growth with water supply-related climate variables in the summer is in accordance with the species’ adaptation to summer drought. In its region of origin, drought periods of up to six months can occur [30]. At the origin of the seeds of the studied *C*. *libani*, individual trees maintain growth even in a year with a precipitation sum of only 63 mm in the summer (June - August) [25]. Similar results of persistent growth during drought periods were found in ecophysiological studies of *C*. *libani* conducted by [24]. During drought periods, transpiration is primarily kept up through water stored in the stem tissues [81, 88, 89]. Furthermore, the high water-holding capacity of the fine soils between the cracks of limestone blocks in its region of origin [90] are likely to compensate for the lack of precipitation. Especially on such soils, *C*. *libani*’s deep-reaching taproot or cordate root system [31] can draw upon water stored in deeper soil layers. Additionally, such root systems enable conifers to exploit spring precipitation better [91]. The avoidance of drought stress induced by water deficits through an increased vertical root penetration was also observed for *Abies alba* [92].

Heat-related climate variables correlated negatively with radial growth of *C*.* libani* during the vegetation period and especially in August. Generally, growth of tree species increases with temperature [93], and cambial activity often requires a temperature threshold to be exceeded. For *C*. *libani*, [94] found that threshold to be a daily minimum temperature of 0°C and a daily mean air and stem temperature of 5°C. However, high temperatures are a major limiting factor for plant productivity [95]. Hence, it is not surprising that significant negative correlations with heat sum were found. In its natural stands in Turkey, [26, 94] also detected a significant negative correlation between daily stem radius increment and temperature variables between June and August and between daily stem radius variation and T_max_ during the main growing period. Overall, *C*. *libani* from Turkey grows well under temperatures at the study site and thus also at similar sites in many parts of Central Europe. However, extremely high temperatures during the vegetation period may reduce radial increment.

Radial growth of *P*. *abies* was reduced by heat during the vegetation period, and especially in July and August. As for *C*. *libani*, high temperatures can be expected to limit its productivity [95]. In combination with its shallow root system and weak stomata-control [9, 70], the aggravation of drought stress, caused by high temperatures, seems to be a crucial factor for *P*. *abies* during the growing season. In accordance, [9] found high temperatures in June to limit radial growth of the native conifer species. Moreover, according to species-specific climate distribution diagrams (german: "Klimahüllen"), *P*. *abies* already grows at the upper margin of the mean annual temperature amplitude tolerated at a site with an annual precipitation sum like at the study site [96]. Hence, the cultivation conditions in that area are likely to deteriorate with ongoing climate change.

High temperatures during the vegetation period and from July through September were significantly negatively correlated with radial growth of *P*. *sylvestris*. This is likely to be a result of heat-induced reductions of plant physiological processes [97] and the enhancement of drought stress due to a high evapotranspiration demand, leading to stomatal closure [85, 86]. Furthermore, very high respiration rates caused by high temperatures can cause a negative mass balance of *P*. *sylvestris* [71], which might explain its even stronger susceptibility to heat stress than that of the other species. Further studies conducted on radial growth in *P*. *sylvestris* emphasise a negative impact of (maximum) temperature in June ([98]; the authors show this negative effect at the example of two German temperate forest national parks) and from June through August [76, 85].

No significant correlations of radial growth of *C*. *libani* with cold-related climate variables could be found, indicating frost-tolerance of the species. Accordingly, in its region of origin at high elevation, temperatures of -35°C can occur [19] and the species is adapted to cold winters and heavy snowfall [24, 33]. In a study about frost tolerance of 27 tree species cultivated in the Ecological-Botanical Garden, [99] showed that *C*. *libani* tolerates at least -24°C in February, reaches its maximum frost tolerance already in November and is able to maintain it until March.

Even though *C*. *libani* is well adapted to summer drought, the growth period of its natural stands is shorter [25], as growth cessation is highly influenced by water availability, and water reserves are usually depleted at the end of the summer [94]. Hence, despite summer drought adaptation of *C*. *libani*, water availability is a limiting factor of growth rates [100]. Sufficient water supply, especially at the beginning of the vegetation period, is crucial and evenly distributed precipitation throughout the year is favourable for strong radial growth of this species. Overall, summer droughts, intensified through rising temperatures, will make the cultivation of *P*. *abies* difficult at a site in Central Europe with a comparable setting as the study area in the future [8, 96, 101]. Altogether, opinions on *P*. *sylvestris*’ overall drought tolerance differ, whereas e.g., [91, 102] claim such tolerance, several recent studies do not confirm this hypothesis [73, 76, 103, 104].

However, the generalizability of the results for entire Central Europe is unclear, as this study was limited to a single site. Furthermore, using radial growth instead of volume increment neglects the distribution of incorporated resources to other parts of a tree than the stem at breast height. Hence, growth depressions in certain years may be over-estimated. Nonetheless, obtaining tree cores is a non-destructive method of data collection and enables comparisons across studies, as this method is commonly used to investigate climate-growth relationships. Additionally, a solid long-term climatic trend is less likely to be visible in a short time series of rather young trees like the ones analysed. Interestingly, resilience values differed severely across studies. This demonstrates that the severity of climatic stress, different calculations methods for the response indices, and local site conditions have to be taken into account when comparing stress tolerance of a species across studies.

## Conclusion

This study is, to our knowledge, the first study to compare growth responses to climatic stress events of non-native *Cedrus libani* with the native conifer species *Picea abies* and *Pinus sylvestris* in Central Europe. In summary, radial growth of *C*. *libani* subsp. *stenocoma* was less impacted by summer droughts compared to native conifers. On average, the species was more resilient to climatic stress events, confirming the main hypothesis of this study, that *C*. *libani* reacted less to climatic stress events than co-occurring *P*. *abies* and *P*. *sylvestris*. Climatic stress tolerance of *C*. *libani* combined with its valuable wood properties [19] leads to the conclusion that *C*. *libani*, and especially its provenance originating from the Taurus Mountains, should be considered a potential novel tree species for forestry in Central Europe. Due to its high light demand and susceptibility to competition [18, 25], a mere substitution of *P*. *abies* with *C*. *libani*, is not recommended. The pursuit of establishing stable, species-rich mixed forests should rather be essential [10, 13, 14]. Additionally, an introduction of the novel conifer species into protected areas, such as national parks or Natura 2000 sites should be adverted, as impacts are unforeseeable. Instead, cultivation in open spaces (for example, after clear-cuts due to bark beetle infestations), in mixture with other, mainly native, tree species, could present an option. This would furthermore take advantage of the natural regeneration of autochthonous trees. Any cultivation, however, would be based on the premise that *C*. *libani* is a tree species harmless to the endemic flora and fauna and remains largely insensitive to biotic and abiotic damages. Apart from its growth and climatic stress tolerance on other sites in Central Europe, particularly impacts on native flora and fauna need to be addressed in future studies.

## Supporting information

S1 File (DOCX)

S2 File (DOCX)

S3 File (DOCX)

S4 File A detailed description of the different climatic characteristics of each stress event. (DOCX)

S1 Table Statistical parameters Ar1, calculated for the radial growth chronologies, and Rbar as well as EPS, calculated for the standardised growth index chronologies, for *C*. *libani*, *P*. *abies* and *P*. *sylvestris*. (DOCX)

S1 Fig Average relative radial growth of *C*. *libani* (blue), *P*. *abies* (red) and *P*. *sylvestris* (yellow) (a) in the year before [Pre SE (1)], of and after [Post SE (1)] a climatic stress event (SE) and (b) on average in the two years before [Pre SE (2)]/after [Post SE (2)] and in the year of a climatic stress event. Average relative radial growth was calculated as the median of relative radial growth for (a) the SEs 2003, 2012, 2015 and 2018 and (b) the SEs 2003, 2012 and 2015.
(TIF)

S1 Data (ZIP)
