# Supplementary material for: Impact of climatic conditions on radial growth of non-native Cedrus libani compared to native conifers in Central Europe
Source: PLoS One. 2023 May 12;18(5):e0275317. doi: 10.1371/journal.pone.0275317 (PMC10180601; doi:10.1371/journal.pone.0275317)
Supplement: S2 File — (DOCX) [file pone.0275317.s002.docx]

Supporting Information

$$e_{s}=6.11\times e^{\frac{17.62 \times T}{(234.12+T)}}$$

Equation S2

e_s_ [hPa]: saturation vapour pressure
