# Supplementary material for: Impact of climatic conditions on radial growth of non-native Cedrus libani compared to native conifers in Central Europe
Source: PLoS One. 2023 May 12;18(5):e0275317. doi: 10.1371/journal.pone.0275317 (PMC10180601; doi:10.1371/journal.pone.0275317)
Supplement: S3 File — (DOCX) [file pone.0275317.s003.docx]

Supporting Information

$$e_{a}= \frac{RH}{100} \times e_{s}$$

Equation S3

RH: relative humidity

e_s_ [hPa]: saturation vapour pressure

e_a_ [hPa]: actual vapour pressure
