# Supplementary material for: Impact of climatic conditions on radial growth of non-native Cedrus libani compared to native conifers in Central Europe
Source: PLoS One. 2023 May 12;18(5):e0275317. doi: 10.1371/journal.pone.0275317 (PMC10180601; doi:10.1371/journal.pone.0275317)
Supplement: S4 File — (DOCX) [file pone.0275317.s004.docx]

Supporting Information

**S1 Results:** A detailed description of the different climatic characteristics of each stress event.

**Climatic characteristics of detected stress events**

Each stress event exhibited different climatic characteristics. The overall water supply during the rather cold (T_mean_ ≤ the 25th percentile) PW of 2003 was high (PPT ≥ the 90th and nrd (= number of rain days with PPT > 1 mm ) ≥ the 75th percentile) but got increasingly less over time. The VP in 2003 started off rather cold in April (nfd ( = number of frost days with T_min_ ≤ 0 °C) ≥ the 75th percentile) and then warm and wet in May (T_mean_ and PPT ≥ the 75th percentile). For the rest of the VP temperatures were warm - hot, particularly in June and August (T_mean_ ≥ the 90th percentile). Additionally, from June through September, there was a lack of precipitation (PPT and dmi ≤ the 10th/25th percentile), stringent in June and especially in August. Apparently, precipitation at the beginning of the VP was not enough to compensate for its lack in the preceding (winter) months, as spei3 values remained < -1 (May, July, September)/< -1.5 (April, June, August) for the entire VP, indicating a persistent moderate/severe drought which reached its culmination and became extreme in August (spei1 < -2).

Concerning temperature and precipitation, the PW of 2012 (10/2011 - 03/2012) could for its first part be classified as average, but turning towards its end cold (nid (= number of ice days with T_max_ ≤ 0 °C) ≥ the 90th percentile in February)- and then warm (T_mean_ ≥ the 75th percentile in March) and dry (PPT and nrd ≤ the 10th/25th percentile). This lack of precipitation (PPT and dmi ≤ the 10th/25th percentile) persisted through May, accompanied by high temperatures in that same month (T_mean_ ≥ the 90th percentile). This led to a persistent severe drought in spring (spei3 < -1.5 in April and May), which affected water supply through June (spei3 < -1.0). The rest of the VP could be characterised as average regarding temperature and precipitation, except for August, where temperature increased and precipitation decreased again to values notably above/below average.

The PW of 2015 (10/2014 - 03/2015) was overall very mild (T_mean_ ≥ the 75th percentile) and rather dry (PPT and nrd ≤ the 25th percentile). From March through June, average values for temperature and precipitation could be found for the VP. This changed for the entire rest of the vegetation period, where heat-related climate variables increased to values ≥ the 90th percentile ('number of hot days with T_max_ ≥ 30 °C'  and 'heat sum [°C] of daily T_mean_ > 20 °C'  in July - August; T_mean_ in August - September) and PPT values decreased to ≤ the 10th/25th percentile for all months. This was accompanied by high VPD and small dmi values. spei3 values were < -1.0 (July and August)/ < -1.5 (September), ascribing 2015 a persistent severe drought during its summer.

Regarding temperature and precipitation, the PW of 2018 (10/2017 - 03/2018) could overall be characterised as average. On a monthly scale T_mean_ changed from relatively high to quite low during the course of the winter. Through the entire VP, mean temperature values exceeded the 75th percentile (≥ the 90th percentile in April, May, August), and except for June, VPD values the 90th percentile. Precipitation was initially average but became scarce in June and especially July and August. A persistent severe drought (spei3 < -1.5) could be detected already from April on, lasting for the entire VP through September and reaching its culmination and becoming extreme in July and August (spei3 < -2.0).
