# Supplementary material for: Impact of climatic conditions on radial growth of non-native Cedrus libani compared to native conifers in Central Europe
Source: PLoS One. 2023 May 12;18(5):e0275317. doi: 10.1371/journal.pone.0275317 (PMC10180601; doi:10.1371/journal.pone.0275317)
Supplement: S1 Table — (DOCX) [file pone.0275317.s005.docx]

Supporting Information

**S1 Table**

|  | *C. libani* | *P. abies* | *P. sylvestris* |
| --- | --- | --- | --- |
| **Ar1** | 0.60 | 0.55 | 0.57 |
| **Rbar** | 0.39 | 0.48 | 0.45 |
| **EPS** | 0.94 | 0.92 | 0.90 |

Ar1 = mean first-order autocorrelation, Rbar = mean inter-series correlation, EPS = expressed population signal.
