# Supplementary figures and images for: Impact of climatic conditions on radial growth of non-native Cedrus libani compared to native conifers in Central Europe
Source: PLoS One. 2023 May 12;18(5):e0275317. doi: 10.1371/journal.pone.0275317 (PMC10180601; doi:10.1371/journal.pone.0275317)

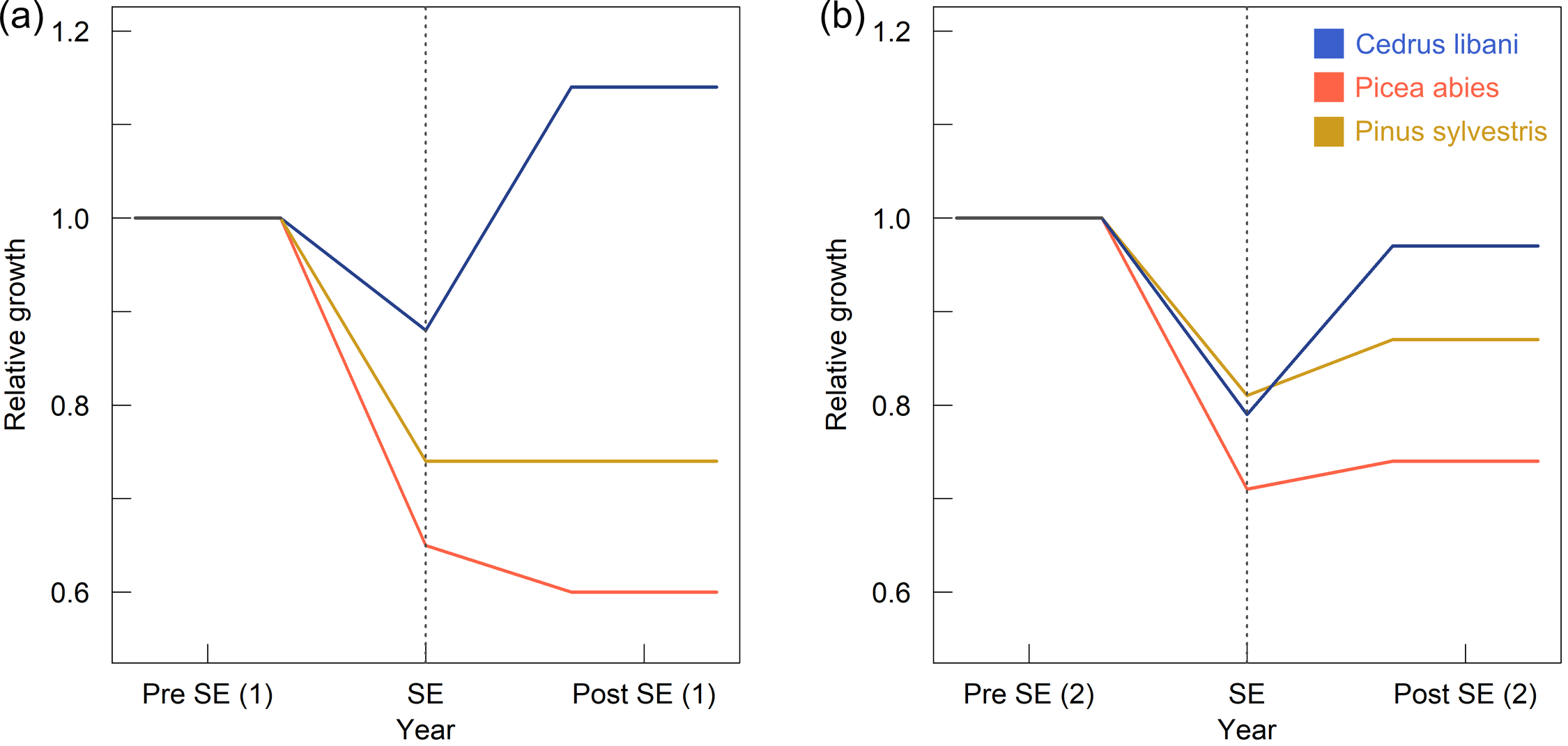

Supplement: S1 Fig — Average relative radial growth of C. libani (blue), P. abies (red) and P. sylvestris (yellow) (a) in the year before [Pre SE (1)], of and after [Post SE (1)] a climatic stress event (SE) and (b) on average in the two years before [Pre SE (2)]/after [Post SE (2)] and in the year of a climatic stress event. Average relative radial growth was calculated as the median of relative radial growth for (a) the SEs 2003, 2012, 2015 and 2018 and (b) the SEs 2003, 2012 and 2015. (TIF) [file pone.0275317.s006.tif]
