# Supplementary material for: Impact of climatic conditions on radial growth of non-native Cedrus libani compared to native conifers in Central Europe
Source: PLoS One. 2023 May 12;18(5):e0275317. doi: 10.1371/journal.pone.0275317 (PMC10180601; doi:10.1371/journal.pone.0275317)
Supplement: S1 Data — (ZIP) [file pone.0275317.s007.zip › tree core measurements/treecore measurement data.docx]

Treecore measurement data

BAYR_xxxx_1/2 : two measurements of a single tree core

BAYR_*species*_undated : all undated tree core measurements (arythmetical average of the two measurements per tree core) per species

all tree cores_*species*_dated : all dated tree core measurements (arythmetical average of the two measurements per tree core) per species
